# Adenoviral Delivery of the VEGF and BMP-6 Genes to Rat Mesenchymal Stem Cells Potentiates Osteogenesis

**DOI:** 10.1155/2013/737580

**Published:** 2013-02-26

**Authors:** Jesse Seamon, Xiuli Wang, Fuai Cui, Tom Keller, Abhijit S. Dighe, Gary Balian, Quanjun Cui

**Affiliations:** Orthopaedic Research Laboratories, Department of Orthopedic Surgery, University of Virginia School of Medicine, P.O. Box 800159, Charlottesville, VA 22908, USA

## Abstract

The combined delivery of mesenchymal stem cells (MSCs), vascular endothelial growth factor (VEGF), and bone morphogenetic protein (BMP) to sites of bone injury results in enhanced repair compared to the administration of a single factor or a combination of two factors. Based on these findings, we hypothesized that coexpression of VEGF and BMP-6 genes would enhance the osteoblastic differentiation of rat bone-marrow-derived stem cells (rMSCs) and osteogenesis by comparison to rMSCs that do not express VEGF and BMP-6. We prepared a GFP tagged adenovirus vector (Ad-VEGF+BMP-6) that contained DNA encoding the hVEGF and hBMP-6 genes. rMSCs were transduced with the virus, and the successful transduction was confirmed by green fluorescence and by production of VEGF and BMP-6 proteins. The cells were cultured to assess osteoblastic differentiation or administered in the Fischer 344 rats to assess bone formation. Mineralization of rMSCs transduced with Ad-VEGF+BMP-6 was significantly enhanced over the nontransduced rMSCs. Only transduced rMSCs could induce osteogenesis *in vivo*, whereas Ad-VEGF+BMP-6 or nontransduced rMSCs alone did not induce osteogenesis. The data suggests that the combined delivery of MSCs, VEGF, and BMP-6 is an attractive option for bone repair therapy.

## 1. Introduction

 Bone morphogenetic proteins (BMPs) are members of the TGF-beta superfamily that possess a number of physiologic activities including the maintenance and stimulation of osteoblast differentiation [1]. The BMPs exert their effects on target tissues by binding to two types of serine/threonine kinase receptors forming a complex that phosphorylates transcription factors referred to as SMADs [2]. SMADs then act at the genomic level to alter the expression of proteins by target cells [1–3]. While BMPs have been shown to have a wide range of physiologic activities, the primary focus in the orthopaedic literature has been on their osteogenic properties that relate to bone formation *in vitro* and *in vivo*, as well as the ability of BMPs to promote skeletal repair and healing of critical sized bone defects [2, 4–28]. Several studies have attempted to determine the osteogenic potential of individual BMPs in comparison to one another [2, 12, 14, 17–19, 29]. Studies by Kang et al. [14], Li et al. [18], and Luu et al. [19] have shown that BMP-6 and BMP-9 possess superior osteogenic potential compared to BMP-4 and BMP-7 and at least equal to the osteogenic potential of BMP-2. Investigations by Vukicevic and Grgurevic [26] and Ebisawa et al. [2] have shown that BMP-6 is a more potent inducer of osteogenesis than BMP-7. Currently, only BMP-2 and BMP-7 are approved by the FDA for human use [5].

Vascular endothelial growth factor (VEGF) is an angiogenic growth factor that is expressed by endothelial cells [3]. Angiogenesis is an important part of the bone repair process and is crucial for the supply of nutrients to developing cells along with incoming new cells that contribute to the process. VEGF has gained considerable interest in orthopaedics based on a number of reports that VEGF promotes neovascularization and growth of bone in animal models and during distraction osteogenesis [7, 22, 23, 25, 30–33]. 

A bone graft substitute with both angiogenic and osteogenic properties is desirable in the field of orthopaedics. Early angiogenesis would promote revascularization of a facture site and increase the delivery of nutrients, growth factors, and osteoblastic precursors to a fracture thereby enhancing bone repair. The presence of osteogenic factors would provide the stimulus for a high percentage of precursor cells at the fracture site to differentiate and promote bone healing. A number of studies in the orthopaedic literature have demonstrated synergism between BMP-2/BMP-4 and VEGF, and this synergism is pronounced in the early phases of bone healing [9, 11, 13, 21–25, 32]. While the majority of the studies have focused on the delivery of BMP-2 and BMP-4 with VEGF, there have been few reports on the delivery of VEGF and BMP-6 despite several studies which suggest that BMP-6 is one of the more potent BMPs for osteogenesis [2, 14, 18, 19, 26]. A recent study in our laboratory [9] with a cloned mouse osteoprogenitor cell (D1) has shown that transfection with plasmids containing both BMP-6 and VEGF produced more bone than D1 cells that were transfected with plasmids containing only the BMP-6 or the VEGF gene. However, from a practical point of view, freshly prepared bone marrow cells are more clinically relevant. Similarly, transient transfection of MSCs using plasmid vectors encoding VEGF or BMP gene can express the therapeutic protein only for a limited time in contrast to adenoviral transduction of MSCs that can produce VEGF and BMP proteins in abundant quantity for a prolonged time period. Therefore, in our present study, we created an adenovirus construct containing both the VEGF and the BMP-6 genes, and, through transduction of a mixed cell population obtained from bone marrow blow outs of the Fisher 344 rats, we demonstrated the effects of this combination on matrix mineralization *in vitro* and osteogenesis *in vivo*. 

## 2. Materials and Methods

### 2.1. Animal Care

The work in this study, utilizing the Fisher 344 rats, was performed under the protocol guidelines of the University of Virginia's Animal Care and Use Committee, IACUC Study no. 3701. The policies of the Animal Care and Use Committee were monitored and maintained during all testing and handling of animals.

### 2.2. Adenovirus Construct (Ad-VEGF+BMP-6)

The first step was to clone the human VEGF (hVEGF), IRES, and human BMP-6 (hBMP-6) genes into a TOPO-TA vector. The complete human 589 base pair segment of VEGF cDNA (GenBank Access no. AF486837) was PCR amplified from a plasmid containing the human VEGF gene [9] using the primers.

Forward: 5′-AT*CGATCG*ATGAACTTTCTGCTGTCTTGG-3′

Reverse: 5′-C*CTCGAG*TCACCGCCTCGGCTTGTCACATCTGC-3′

An IRES sequence was PCR amplified from the shuttle vector pShuttle-IRES-hrGFP-1 (Stratagene, La Jolla, CA). The 1554-base pair human BMP-6 gene (GenBank Access no. NM_001718) was amplified from a plasmid expressing BMP-6 (Origene, Rockville, MD) using the primers. 

Forward: 5′-GCTAGCATGCCGGGGCTGGGG-3′

Reverse: 5′-GATATCTTAGTGGCATCCACAAGCTCTTACAAC-3′

Each cDNA segment was cloned into the TOPO-TAvector (Invitrogen Corporation, Carlsbad, CA). The clones were confirmed by restriction enzyme digestion and complete DNA sequence analysis. The next step was to subclone the hVEGF, hBMP-6, and IRES sequences into a pShuttle-IRES-hrGFP-1 plasmid. The hVEGF gene was cloned into the pShuttle-IRES-hrGFP-1 plasmid to generate pShuttle-VEGF-IRES-hrGFP-1 using the restriction enzymes PvuI and XhoI. Next, the IRES was subcloned into the pShuttle-hVEGF-IRES-hrGFP plasmid using the restriction enzymes EcoRV and PvuI to generate the pShuttle-IRES-VEGF-IRES-hrGFP sequence. Lastly, BMP-6 was subcloned into the plasmid using the NheI and EcoRV restriction enzymes to generate pShuttle-hBMP-6-IRES-hVEGF-IRES-hrGFP. DNA sequencing and restriction enzyme digestion were used to confirm successful subcloning of the hBMP-6-IRES-hVEGF sequences into the plasmid.

We then subcloned the hVEGF, IRES, and hBMP-6 genes into a pAdEasy-1 vector. The pShuttle-hBMP6-IRES-hVEGF-IRES-hrGFP-1 (pShuttle-VB6) construct was linearized using Pme I and was purified using a PCR Purification kit (QIAGEN Sciences, MD, USA). The purified DNA fragment was dephosphorylated using alkaline phosphatase for 30 minutes at 37°C. Hundred *μ*L of chemically competent cells (BJ5183, Stratagene) were mixed gently with 1 *μ*g of the linearized, dephosphorylated pShuttle-VB6 and 1 *μ*L of pAdEasy-1 circular and supercoiled vector (100 ng/*μ*L) in a microcentrifuge tube on ice for 30 minutes. The tube was then transferred into a 42°C water bath for 90 seconds and then put back on ice for 2 minutes. 1 mL of sterile LB broth was immediately added into the tube. The cell suspension was transferred into a sterile 14 mL polypropylene round-bottom tube and mixed at 37°C for 1 hour at 250 rpm/minute. The cells were plated onto LB-kanamycin plates and grown overnight at 37°C. Over forty well-separated colonies were picked and transferred into 5 mL of LB-kanamycin broth. The cultures were grown overnight at 37°C, and DNA was extracted from individual liquid cultures. The final construct was referred to as pAd-hBMP6-IRES-hVEGF-IRES-hrGFP-1 (pAd-VEGF+BMP-6) and linearized by digestion with Pac I.

 Next, AD-293 packaging cells were transfected with Ad-VEGF+BMP-6 to prepare primary virus stock. Lipofectamine 2000 reagent was used to transfect AD-293 cells with the linearized Ad-VEGF+BMP-6. One day before transfection, 1 × 10^6^ AD-293 cells were grown in 2 mL DMEM growth medium in a 6-well tissue culture plate without antibiotics so that the cells become 90–95% confluent at the time of transfection. Transfection complexes were prepared as follows: (a) 4 *μ*g of Pac I linearized Ad-VEGF+BMP-6 DNA was diluted in 250 *μ*L DMEM-high glucose medium without serum by mixing gently; (b) 10 *μ*L of Lipofectamine 2000 was diluted in 250 *μ*L of DMEM-high glucose medium, and the reagent was incubated for 5 minutes at room temperature; (c) the diluted DNA and diluted Lipofectamine 2000 (total volume 500 *μ*L) were mixed together gently and incubated for 20 minutes at room temperature. The cells were washed twice with PBS, and 500 *μ*L of complex was added to each well containing the cells. After gentle mixing, the cells were incubated at 37°C in a CO_2_ incubator for 6 hours, and 1 mL growth medium was added to each well. The cells were further incubated for 24 hours at 37°C. After 24 hours, the growth medium was replenished and the plates with transfected cells were incubated for 10–14 days. The cells were harvested by adding 0.5 mL PBS to each well. The cell suspension was transferred to a 1.7 mL screw-capped microcentrifuge tube and freezed/thawed four times by alternating the tubes between −80°C and 37°C. Cellular debris was collected by microcentrifugation at 12,000 ×g for 10 minutes at room temperature. The supernatant (primary virus stock, Ad-VEGF+BMP-6) was transferred to a fresh screw-capped microcentrifuge tube and stored at −80°C. The plaque forming units of the virus stock were measured by infecting AD-293 cells and counting the number of green fluorescent cells. The virus stock was also tested by PCR to confirm the presence of the hVEGF and hBMP-6 genes.

### 2.3. Isolation of Bone-Marrow-Derived Stem Cells or Mesenchymal Stem Cells (MSCs) from the Fisher 344 Rats

 Eight male Fisher 344 rats were euthanized. Their bilateral tibias and femurs were removed under sterile conditions, and the metaphyseal regions were cut off with a small blade circular saw (Fine Science Tools, Foster City, CA). An 18-gauge needle attached to a 10 mL syringe was used to “blow out” the bone marrow from the bones into 10 mL DMEM culture medium, and 2 mL of the suspension was transferred to a 75 cm^2^ culture flask with 10 mL of DMEM, penicillin (100 U/mL), streptomycin (100 *μ*g/mL), and 15% FBS. The medium was changed 48 hours later, and subsequently the media were refreshed every 72 hours. The mixed marrow cells used in this experiment were from passages 4–6.

### 2.4. The Transduction of the Fisher 344 Rat Bone-Marrow-Derived Stem Cells (rMSCs)

 rMSCs were maintained in cell culture under standard conditions (37°C with 5% CO_2_). Twenty-Four hours before infection, cells were seeded at 50,000 cells/well in a 24-well plate and allowed to adhere overnight. Infection was performed with Ad-VEGF+BMP-6 at 100 MOI. The Ad-VEGF+BMP-6 virus was thawed on ice and diluted in a microcentrifuge tube with DMEM low glucose medium and 250 *μ*L of fluid was added to each well. Cells in each well were preincubated with 1 mL serum-free DMEM low glucose medium for 20 minutes. The medium was aspirated from each well and the 24-well plate was placed in an incubator at 37°C for 3 hours with gentle rocking every 15 minutes. After 3 hours, an additional 1 mL of culture medium was added to each well and incubated for another 24 hours. Medium containing the virus was replaced with 1 mL of complete culture medium and the cells were incubated for an additional 24 hours. Fluorescence microscopy was used to determine effective infection of the cells by visualizing green fluorescence within the cells.

### 2.5. Subcutaneous Injection of rMSCs and the Ad-VEGF+BMP-6

Three different cell and Matrigel (Becton, Dickinson and Company, San Jose, CA) compositions were prepared for administration in three groups of rats by subcutaneous injection (Table 1). Group 1 consisted of Matrigel mixed with Ad-VEGF+BMP-6 at 10^7^ pfu per 100 *μ*L, with no rMSCs. Group 2 represented noninfected rMSCs at a concentration of 2 × 10^5^ cells per 100 *μ*L and Ad-VEGF+BMP-6 at 10^7^ pfu per 100 *μ*L mixed with Matrigel. Group 3 consisted of rMSCs infected with Ad-VEGF+BMP-6 mixed with Matrigel at a concentration of 2 × 10^5^ cells per 100 *μ*L. There were 2 Fisher 344 male rats in each of the previously described groups (Table 1). Each rat was shaved, and the dorsum of the rat was prepped with betadine and ethanol. A 1 mL syringe was used to inject 100 *μ*L subcutaneously at 4 locations along the dorsum of each rat. Successful injection was confirmed by visualizing a pellet of the Matrigel solution under the subcutaneous tissue.

### 2.6. BMP6 and VEGF Protein

To analyze BMP6 and VEGF production, Ad-VEGF+BMP-6 infected rMSCs were seeded in 24-well plates at 50,000 cells/well in DMEM 1 day before transduction. Over a period of 28 days, 1 mL of medium per day was collected and replaced with fresh medium. The collected medium was stored at −80°C until analysis of the BMP-6 (RayBiotech, Inc., Norcross, GA) and VEGF protein with ELISA (Invitrogen Corporation, Carlsbad, CA). Optical densities were measured at 450 nm using a VERSAmax microplate reader (Molecular Devices, Sunnyvale, CA).

### 2.7. Von Kossa Stain

A von Kossa stain kit (Master Tech. Inc., Lodi, CA) was used to detect mineralization *in vitro* in the Ad-VEGF+BMP-6 infected rMSCs. Cells were selected and rinsed twice with distilled water, placed in a 5% (wt/vol) silver nitrate solution, exposed to sunlight for one hour, washed with distilled water, and placed in 5% sodium thiosulfate solution for 3 minutes. After thorough rinsing with distilled water, the cells were stained with a nuclear fast red stain for 5 minutes, washed again with distilled water, and examined on a microscope at a magnification of 100x. Staining with von Kossa was performed on cells in culture at 2 and 3 weeks.

### 2.8. Harvesting of Subcutaneous Implants

At 3 weeks and 4 weeks, one rat from each group was euthanized in a CO_2_ chamber. A 15-blade scalpel was used to make an incision along the dorsum of the rat from the base of the skull to the proximal portion of the tail. Metzenbaum scissors were used to carefully dissect out the subcutaneous tissue in order to localize the region of the implanted Matrigel. The pellet was identified and removed en bloc with the surrounding soft tissue. The harvested bone pellet was then placed in a 21 mm diameter sterile test tube containing a solution of 4% paraformaldehyde in PBS.

### 2.9. MicroCT Analysis

High resolution X-ray computed tomography with image-based 3D reconstructions allows for quantification of bone volume on a Viva40 Scanco Micro CT instrument (Scanco Inc., Switzerland) that allows for full three-dimensional reconstructions of biomaterial scaffolds and mineralized tissues, measuring up to 38 mm in diameter and 70 mm in length at a maximum resolution of 6 microns. The previously harvested subcutaneous implants from each group were placed into the *μ*CT instrument in a 21 mm diameter test tube, with foam surrounding each specimen to prevent vibration during the scan. Scans were performed in the axial plane with a slice increment of 21 *μ*m at 45 kvp. Following completion of the primary scan, the raw images were converted to DICOM files and analyzed using MIMICS ver 3.1 software (Materialise, Plymouth, MI). Thresholds were selected for each sample after all soft tissues were removed so that only boney tissue remained visible followed by 3D reconstructions and calculations of bone volume. To prevent overestimating bone volume, we used the preloaded BONE CT threshold on the software with fine adjustments for each sample to ensure that no soft tissue was visible. 

## 3. Results

### 3.1. Ad-VEGF-BMP-6 Efficiently Transduces the rMSCs

**Figure 1:**
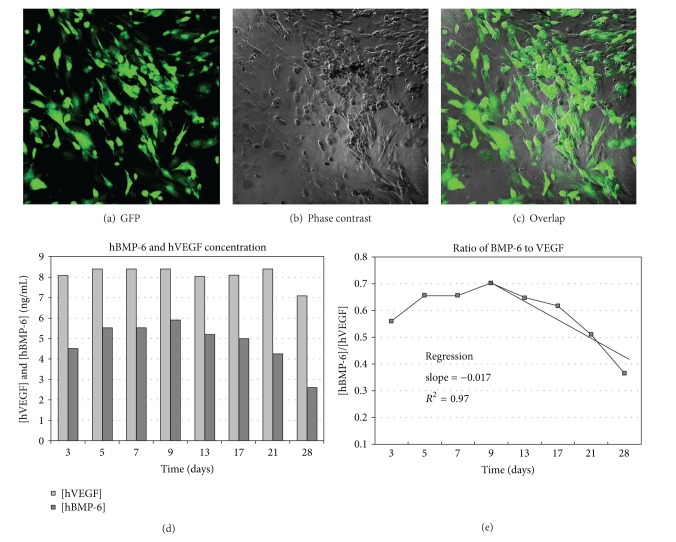
Expression of BMP-6 and VEGF in rMSCs. (a, b, and c): Microscopy of rMSCs infected with adenovirus containing genes viewed in dark field for GFP fluorescence (a), bright field by phase contrast (b), and overlapping dark and bright fields (c). (d) Concentration of growth factors (ng/mL) in medium of cells infected with Ad-VEGF+BMP-6 and cultured for 4 weeks. The concentration of hVEGF is maintained nearly constant, while the hBMP-6 peaks at 9 days and then decreases steadily. (e) The ratio of hBMP-6 to hVEGF produced by infected cells over a 28-day period in culture. The concentration of hVEGF is consistently greater than hBMP-6 at all time points. Regression analysis shows that the ratio diminishes by 0.017 per day after 9 days (2.5% per day).

 The Ad-VEGF+BMP-6 construct contained the GFP sequence to evaluate the infected cells microscopically. Infection of the rMSCs MMCs was evident by the fluorescence of the GFP in the dark field image (Figure 1(a)). Superimposing this image over the image of cells viewed in bright field (Figure 1(b)) further revealed the efficiency of transduction with Ad-VEGF+BMP-6 (Figure 1(c)).

 Culture medium was collected from the cells at 3, 5, 7, 9, 13, 17, 21, and 28 days and analyzed for hVEGF and hBMP-6 (Figure 1(d)). VEGF production remained elevated throughout the course of the experiment at a concentration greater than that of BMP-6. Production of BMP-6 peaked at around 9 days and decreased gradually to approximately 50% by day 28; however, a significant amount of BMP-6 was still present in the culture medium at this time point. The ratio of BMP-6 to VEGF at each time point up to 28 days in culture shows values between 0.4 and 0.7 indicating that the cells produced approximately twice the amount of VEGF compared to BMP-6 (Figure 1(e)). A regression analysis after 9 days shows a diminished ratio of BMP-6 to VEGF of 0.0174 per day (2.5% decrease in the ratio per day), with chi-square (*R*) agreement equal to 0.97.

### 3.2. Coexpression of VEGF and BMP-6 Genes in rMSCs Enhances Mineralization *In Vitro*

**Figure 2:**
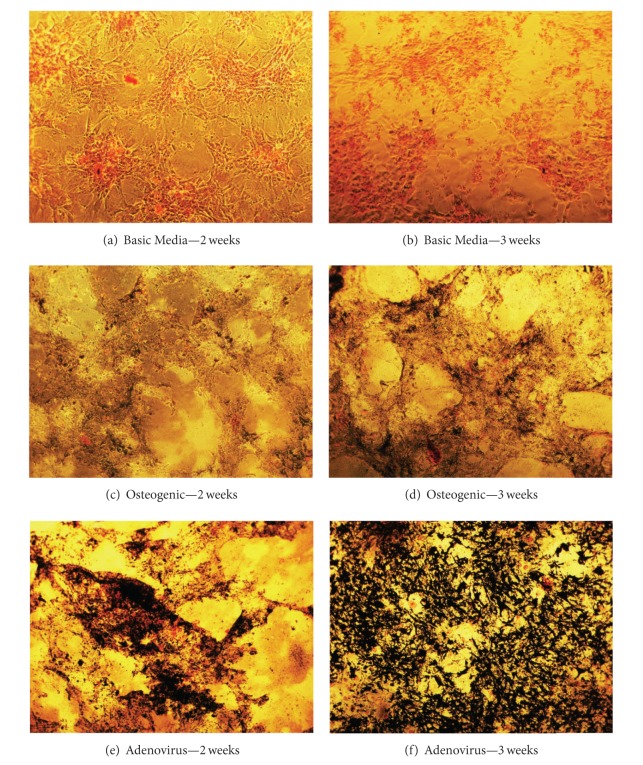
Von Kossa staining of cells in culture at 2 and 3 weeks. Black areas reflect the presence of mineral in the cultures. No mineralization is seen in the basic medium at 2 weeks (a) and 3 weeks (b). The noninfected rMSCs at 2 weeks (c) and 3 weeks (d) show minimal mineralization. Images of the Ad-VEGF+BMP-6 infected mixed marrow cells at 2 weeks (e) and at 3 weeks (f) showed considerable mineralization.

Mineralization of the Ad-VEGF+BMP-6 transduced rMSCs was detected *in vitro* by staining with von Kossa after the cells were maintained in culture for 2 and 3 weeks (Figure 2). No mineralization was seen in the basal medium. The noninfected rMSCs at 2 weeks and 3 weeks showed minimal mineralization. Images of the Ad-VEGF+BMP-6 transduced rMSCs at 2 weeks and at 3 weeks showed significant mineralization. These observations indicated that while the noninfected rMSCs exhibited minimal osteogenic potential, the same cells showed extensive matrix mineralization in culture after transduction with Ad-VEGF+BMP-6.

### 3.3. rMSCs Transduced with Ad-VEGF+BMP-6 Induce Significant Osteogenesis *In Vivo*, but rMSCs or Ad-VEGF+BMP-6 Alone Fail to Induce Osteogenesis

**Figure 3:**
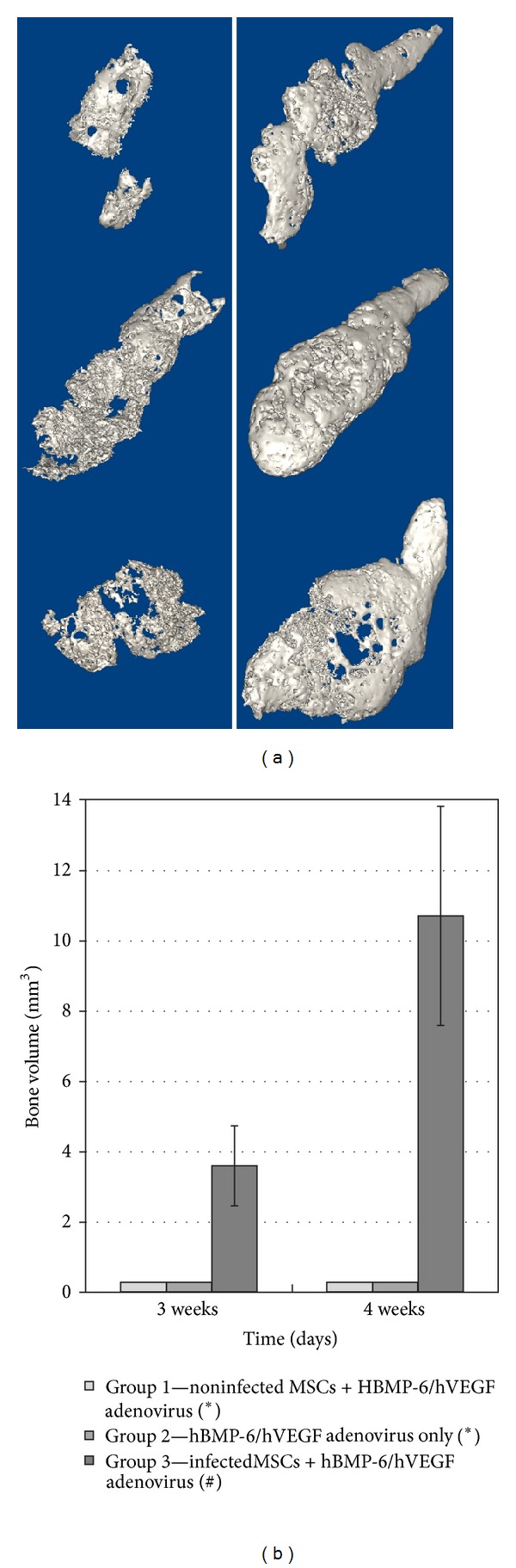
Osteogenesis induced by rMSCs. (a) Reconstructions from axial microCT slices of specimens retrieved from rats injected with Matrigel + cells that were infected with adenovirus (Ad-VEGF+BMP-6). The less porous 3D structure at 4 weeks (right panel) denotes an increase in bone volume as compared to the bone volume at 3 weeks (left panel). (b) Bone volume of the implants retrieved from the rats from 3 groups of subcutaneous (SQ) injections. The bars represent the standard deviations of the means. (∗) trace amounts of tissue estimated with no standard deviation. (#) *P* < 0.05 statistical significance between 3 and 4 weeks.

**Figure 4:**
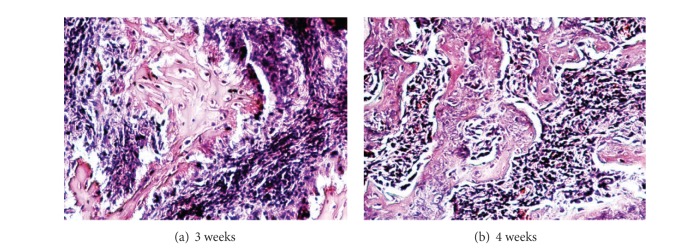
Histology of tissue retrieved at 3 weeks (a) and 4 weeks (b) showing bone formation after injection of Ad-VEGF+BMP-6 infected marrow cells expressing hVEGF and hBMP-6 (stain: H&E, original magnification 60x).

A remarkably small amount of soft tissue was visible at the injection sites in the groups receiving adenovirus construct alone (Group 1) or sites that received noninfected rMSCs (Group 2). By contrast, there was clearly visible bone and soft tissue in the specimens retrieved from sites injected with transduced rMSCs (Group 3) at both 3 and 4 weeks (Figure 3(a)). Bone volume was significantly larger at 4 weeks compared to 3 weeks (Figure 3(b)). A two-tailed *t*-test shows statistical significance (*P* = 0.045) in bone volume between 3 weeks and 4 weeks in Group 3. Histological analysis of the implants of transduced rMSCs showed genuine bone formation at 3 and 4 weeks (Figure 4).

## 4. Discussion

Numerous orthopaedic surgical procedures necessitate the use of bone grafts, from elective spinal fusion to the treatment of open fractures with segmental bone loss and fracture nonunions. Historically, bone grafts have been obtained from allogeneic or autologous sources. Allografts carry a risk of infection, can be slow to incorporate, and may weaken with time. Autografts are usually incorporated effectively but are associated with significant donor site morbidity and are limited in supply. Recent literature has focused on the development of potential bone graft substitutes, and current research has drawn attention to the combination of MSCs, VEGF, and BMPs. 

Several studies have compared the osteogenic potential of individual BMPs relative to one another [2, 12, 14, 17–19, 29]. Notably, Kang et al. [14], Li et al. [18], and Luu et al. [19] reported that BMP-6 and BMP-9 have equal potency compared to BMP-2 and possess more osteogenic potential than BMP-4 and BMP-7. Furthermore, Ebisawa et al. [2] have shown that BMP-6 is 10 times more potent than BMP-7 at promoting the differentiation of C2C12 cells towards osteogenesis. Vukicevic and Grgurevic [26] in their review of multiple studies investigating the role of BMP-6 on osteogenesis highlighted several key features, namely, that BMP-6 is required in significantly lower quantities than BMP-7 for healing critical sized defects in rabbits. Only BMP-6 and BMP-2 are expressed in endochondral bone formation during chondrocyte hypertrophy, and, endochondral bone formation during bone defect repair is impaired in BMP-6 deficient mice [26]. From these observations, they concluded that BMP-6 is the most potent and consistent among the BMPs as a regulator of mesenchymal cell differentiation into osteoblasts [26]. The administration of BMP-6 systemically to osteoporotic rats restored boney architecture, inductive capacity, and trabecular volume of the skeleton [34]. Also, BMP-6 is likely to exert its osteogenic effect through interactions with the IGF-1 and EGF pathways [10]. Metatarsal osteotomies in equines treated with percutaneous injection of BMP-6 in an adenovirus vector carrier 14 days after surgery resulted in faster healing and improved biomechanical properties compared to untreated controls [12]. Injection of BMP-6 via an adenovirus carrier into osteotomy sites in rabbit ulnas 7 days after surgery resulted in faster healing, increased bone volume, and biomechanical properties identical to nonfractured ulnae by 8 weeks [6]. BMP-6 is expressed in osteoclasts in much greater amounts than BMP-2 and BMP-7, and this increased expression may lead to the stimulation of osteoblastic precursor cells thereby having a critical effect in regulating bone homeostasis [35]. BMP-6 is also capable of directly stimulating osteoclastogenesis [36]. Resistance of BMP-6 to the antagonistic activity of noggin has been shown to depend on lysine 60, a critical amino acid residue in the protein, and modification of the residue at this position to lysine imparts resistance to noggin and agonistic activity to BMP-7 [37]. The selection of BMP-6 over BMP-7 or BMP-2 therefore may be rationalized based on the single amino acid substitution and the corresponding resistance to antagonistic activity of noggin osteogenesis. These studies provide compelling evidence that BMP-6 is one of the most potent, if not the most potent, BMP in promoting osteogenesis. In addition, BMP-6 is likely to play a key function in remodeling bone by virtue of its interactions with osteoclasts and, consequently, could be a highly attractive growth factor for use in bone defects where it would promote bone regeneration and appropriate bone remodeling concomitantly. To date, only BMP-2 and BMP-7 are approved by the FDA for human use, and multiple clinical trials have shown them to be efficacious in promoting healing of difficult fractures, open fractures, and boney nonunions [5]. Given the growing body of evidence that supports the potent osteogenic activity of BMP-6, continued investigation will help to further evaluate its role for potential use in the clinical setting.

VEGF is an angiogenic growth factor that is expressed by endothelial cells [3]. Angiogenesis is an integral part of bone healing, as it is required for the supply of nutrients to developing cells as well as new cells that contribute to the healing process. The process of angiogenesis has gained considerable interest in orthopaedics based on a number of studies that demonstrate neovascularization and growth of bone, both of which are mediated by VEGF [7, 22, 23, 25, 30–33]. A soluble receptor used to block the activity of VEGF in mice prevented the formation of new bone by lowering the overall volume and calcification in calluses, suggesting an important role for VEGF in the initial phases of bone repair [25]. Moreover, in the presence of the VEGF antagonists Flt1, BMP-2-mediated bone formation was shown to decrease [22]. In a BMP-2-induced murine model of heterotopic ossification (HO), the formation of new vessels in the region of BMP-2 delivery is the first critical step in ectopic bone formation, and VEGF expression is responsible for this at an early stage [7]. Finally, VEGF plays a critical role during shockwave-induced repair of fractures in rabbits; this observation is supported by diminished healing at a fracture site that is treated with a monoclonal antibody against VEGF [33].

Mounting evidence has implicated both VEGF and BMPs in the process of bone repair, and more attention is now focused on the combined administration of these growth factors. Multiple studies have evaluated the combined administration of BMP-2 or BMP-4 with VEGF and have confirmed the synergistic relationship between these BMPs and VEGF [9, 11, 13, 21–25, 32, 38]. To our knowledge, our laboratory has performed the only study that evaluates the osteogenic effect of a treatment that combines VEGF and BMP-6 [9]. 

We have shown that adenovirus-mediated gene transfer of the hVEGF and hBMP-6 genes to rMSCs can effectively promote osteogenesis both *in vitro* and *in vivo*. Without the administration of the VEGF and BMP-6 genes, the same cell population did not show significant osteogenic properties *in vitro*. Since we already knew that unlike VEGF or BMP-6 alone a combination of VEGF and BMP-6 could significantly enhance expression of osterix and Dlx5 in MSCs [39], we did not test Ad-VEGF or Ad-BMP-6 alone for their ability to enhance mineralization of rMSCs. The observation that subcutaneous injection of the Ad-VEGF+BMP-6 construct without the rMSCs did not result in any appreciable bone formation, as has been reported previously by others [6, 12, 14], suggests that the administration of the virus did not infect the endogenous cells in the host or that the rate of infection of host cells was insufficient to lead to bone formation *in vivo*, in the subcutaneous sites. Consistent with their poor osteogenic ability *in vitro*, nontransduced rMSCs failed to induce osteogenesis *in vivo*. In contrast to nontransduced rMSCs, Ad-VEGF+BMP-6 transduced rMSCs induced significantly greater osteogenesis. We demonstrated earlier [9] that D1 cells, mouse-bone-marrow-derived stem cells, transfected with a plasmid expressing VEGF and BMP-6, induced significantly more osteogenesis in comparison with that induced by nontransfected D1 cells or D1 cells expressing VEGF or BMP-6 alone. Our data is in agreement with our previous findings that coexpression of VEGF and BMP-6 genes enhances osteogenesis induced by MSCs. However, nontransduced rMSCs failed to induce any osteogenesis unlike nontransfected D1 cells. It is likely that the cloned population of D1 cells possesses inherent ability to mineralize that is lacking in freshly isolated rMSCs.

 This study demonstrates that adenovirus-mediated delivery of hVEGF and hBMP-6 is capable of promoting osteogenesis in a relatively nonosteogenic mixed marrow cell population both *in vitro* and *in vivo*. Further study is needed to better characterize the mixed marrow cell population in order to identify the cells that would best serve as the delivery vehicle for the genes and to optimize the VEGF and BMP-6 ratios that are most beneficial for potentiation of bone repair. Lastly, additional studies are needed to evaluate the efficacy of this construct in a bone defect model.

## 5. Conclusion

The infection of the Fisher-344-rat-derived bone marrow with an adenovirus containing the hBMP-6 and hVEGF genes potentiates matrix mineralization by the cells that are cultured *in vitro* and osteogenesis when the cells are injected into ectopic sites *in vivo*. The relative paucity of osteogenic activity in native Fisher 344 rat bone marrow cells suggests that the combination of hBMP-6 and hVEGF genes promotes osteogenic activity in freshly prepared bone marrow cells. Further studies will help determine if the efficacy of such a combination of growth factors will help bone repair in a defect model and elucidate the cell and molecular mechanism whereby the two growth factors promote osteogenesis.

## Figures and Tables

**Table 1:** Experimental groups.

	Injection received	Number of injections
Group 1	Ad-VB6 mixed with Matrigel, no cells	4 SQ injections for 3 and 4 week time points
Group 2	Noninfected MMCs and Ad-VB6 mixed with Matrigel	4 SQ injections for 3 and 4 week time points
Group 3	MMCs infected with Ad-VB6 and mixed with Matrigel	3 SQ injections for 3 and 4 week time points

MMCs: mixed bone marrow cells; Ad-VB6: pAd-Shuttle-hBMP6-IRES-hVEGF-IRES-hrGFP-1; SQ: subcutaneous.
